# The intersection between the oculomotor and hippocampal memory systems: empirical developments and clinical implications

**DOI:** 10.1111/nyas.14256

**Published:** 2019-10-16

**Authors:** Jennifer D. Ryan, Kelly Shen, Zhong‐Xu Liu

**Affiliations:** ^1^ Rotman Research Institute Baycrest Toronto Ontario Canada; ^2^ Department of Psychology University of Toronto Toronto Ontario Canada; ^3^ Department of Psychiatry University of Toronto Toronto Ontario Canada; ^4^ Department of Behavioral Sciences University of Michigan‐Dearborn Dearborn Michigan

**Keywords:** memory, hippocampus, oculomotor control, eye movements, encoding, retrieval

## Abstract

Decades of cognitive neuroscience research has shown that where we look is intimately connected to what we remember. In this article, we review findings from human and nonhuman animals, using behavioral, neuropsychological, neuroimaging, and computational modeling methods, to show that the oculomotor and hippocampal memory systems interact in a reciprocal manner, on a moment‐to‐moment basis, mediated by a vast structural and functional network. Visual exploration serves to efficiently gather information from the environment for the purpose of creating new memories, updating existing memories, and reconstructing the rich, vivid details from memory. Conversely, memory increases the efficiency of visual exploration. We call for models of oculomotor control to consider the influence of the hippocampal memory system on the cognitive control of eye movements, and for models of hippocampal and broader medial temporal lobe function to consider the influence of the oculomotor system on the development and expression of memory. We describe eye movement–based applications for the detection of neurodegeneration and delivery of therapeutic interventions for mental health disorders for which the hippocampus is implicated and memory dysfunctions are at the forefront.

## Graphical abstract

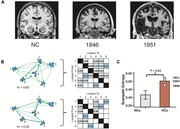

## Introduction

The idea that memory can be revealed through the movements of the eyes is not intuitive. It had long been assumed that the oculomotor (eye movement) system is primarily guided by the physical properties of our visual world (e.g., luminance and contrast), with little to no influence from cognitive processes, such as memory.^1^ Yet, beginning in the 1950s and 1960s, empirical studies made a strong case for memory's influence on where the eyes look and when (i.e., visual exploration).^2^ Russian psychologist Alfred Yarbus^3^ showed that viewers sampled different details of the painting *An Unexpected Visitor* with their eyes depending on the question that was posed.^3^ If the viewer was asked to determine the wealth of the family depicted in the painting, the eyes were directed to the furnishings and wall hangings. If the viewer was asked to give the ages of the people depicted in the painting, eye movements were directed toward the people's faces. Yarbus concluded that the movements of the eyes served to seek information from the visual world, and that visual exploration varies depending on the purpose of the observer and where the requisite information was thought to be found on the basis of prior experience.^3^

**Figure 1:**
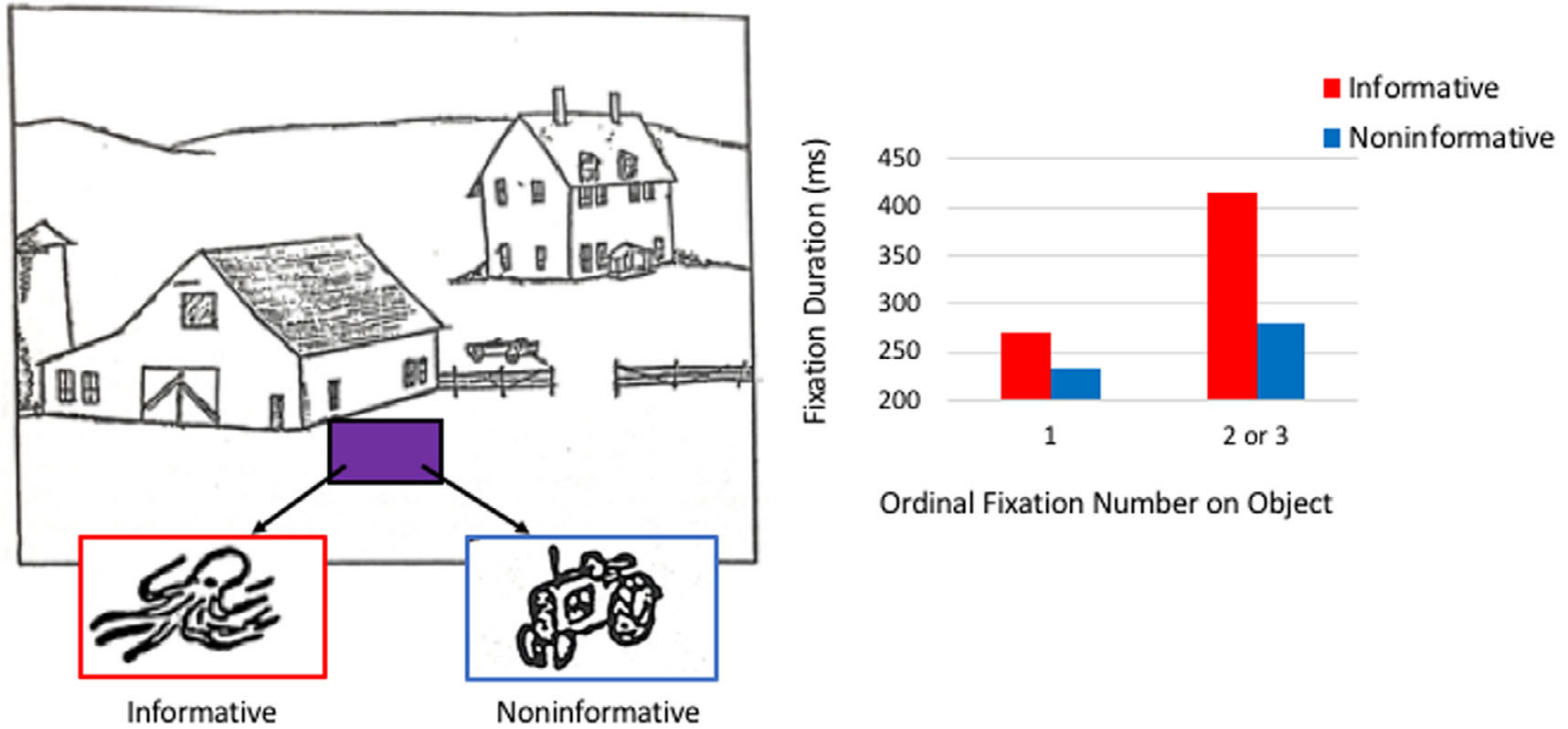
Example stimulus from Loftus and Mackworth.^4^ Viewers were presented with scenes, such as a barnyard scene (left), which contained either an informative (e.g., an octopus) or noninformative (e.g., a tractor) object depending on the meaning of the scene. Fixation durations (right) to informative objects were longer than to noninformative objects, reflecting the additional time needed to extract novel, or unexpected information. Figure adapted from Ref. 4.

This thread of investigation continued through the late 1970s. Loftus and Mackworth^4^ demonstrated that the eyes lingered longer on an object (e.g., an octopus) that was unexpected given the semantics of the surrounding context (a barnyard scene) compared with an object that fit within the meaning of the scene (a tractor; see Fig. 1).^4^ Such findings provided support for Yarbus’ proposition that the eyes seek out informative regions: more information was “embedded” within an unexpected object depending on the knowledge the viewer brought to bear on the experience.

In other studies, recently acquired knowledge was also shown to affect ongoing visual exploration. For example, following the exposure to novel line drawings depicting a scene, such as children sitting at desks in a classroom, viewers’ eye movements were drawn to transformations (i.e., item deletion, size manipulation, and item substitution) that were subsequently made to the pictures;^5^ the eyes seemed to jump ahead to examine these changed regions, suggesting that there was an extraction of information from the periphery that was compared with the information recently stored in memory and, importantly, the evaluation of this comparison process was used to guide further viewing^5^, ^6^ (see Ref. 6 for further discussion).

In the ensuing years, a wealth of evidence followed in the traditions of Yarbus, Loftus, Mackworth, and Parker, among others, to demonstrate the reciprocal link between visual exploration and memory: where we look influences the formation and retrieval of memories, and information retrieved from memory guides our ongoing viewing.^2^ However, despite this evidence, models of oculomotor control have traditionally not considered the influence of memory—nor its underlying neural regions—on visual exploration.

In this article, we review the recent literature that details findings from humans and animal models that use behavioral, neuropsychological, neuroimaging, and computational modeling methods to reveal the intricate link between memory and oculomotor behavior. We describe how information in memory regarding items (e.g., people and objects), such as the arrangement of features within an item, temporal sequences, and the relative spatial positions of items within a broader environment, is used *in the moment* to guide viewing. In addition, we outline how the exchange of information between the hippocampal memory system (including the extended medial temporal lobe (MTL)),^7^, ^8^, ^9^ which critically supports memory for items and their (spatial, temporal, and item‐to‐item) relations, and regions that govern oculomotor behavior is supported by the core architecture of the brain. In doing so, we identify the neural pathways from the hippocampus that influence visual exploration and discuss the nature of information that is exchanged between the systems. Understanding the intersection between the oculomotor and memory systems provides a new conceptualization of an important purpose for eye movements and suggests the need for updated models of oculomotor control in the context of memory and hippocampal function. Knowledge regarding the well‐established links between memory and oculomotor activity will likely further real‐world applications, particularly the development of clinical tools that screen for neurodegeneration involving memory systems, and may provide the treatment for mental health disorders for which dysfunction of the hippocampus and/or the broader MTL is implicated.

## The role of the hippocampus and the extended MTL system in memory

The early work from Loftus and Mackworth,^4^ noted above, revealed the influence of *semantic* memory on viewing. Semantic memory^10^ includes the general knowledge of the world (e.g., Toronto is a city in the province of Ontario) and schemas,^11^, ^12^ which are organized sets of relations interconnected through common elements (e.g., a schema of a farm typically contains a tractor, but not an octopus). Semantic memory can be contrasted with *episodic* memory, which includes the knowledge regarding personally experienced events composed of details such as what, where, and when, along with phenomenological experiences or the awareness of remembering.^13^ Semantic memory and episodic memory can each guide viewing and may compete with one another for oculomotor guidance when recently experienced information conflicts with previously established knowledge.^14^ Semantic memory and episodic memory are subsets of *relational* memory,^7^ which consists of representations regarding the arbitrary associations among items, including item‐to‐item associations, temporal orderings, and the relative spatial arrangements among items.^7^ By definition, semantic memory and episodic memory each reflects sets of relations (e.g., Toronto is the name associated with a particular city; remembering the details of when and how the Toronto Raptors won the National Basketball Association Championship requires storing relations among people, places, and sequences of events). In a similar fashion, memory that can be expressed with concomitant conscious awareness, *explicit* memory, is necessarily relational; in order to overtly comment on the contents of memory regarding a prior episode, relations among a place, time, and the details of an event must have been stored. Memories that are retrieved and influence ongoing performance in the absence of conscious awareness, *implicit* memory, may also be relational to the extent that the successful expression of prior knowledge requires that relations among items had been learned.^15^, ^16^

The hippocampus has a critical role in binding incoming information (including semantic and episodic information and without regard to conscious awareness of the incoming information^17^) into lasting relational representations. The information that is bound by the hippocampus is received from broader MTL structures.^18^, ^19^ The perirhinal cortex (PRC), entorhinal cortex (ERC), and parahippocampal cortex (PHC)—brain regions located within the MTL—support representations of items composed of complex combinations of features,^20^ the configural arrangement of features within and among items,^21^, ^22^, ^23^ and the broader spatial and nonspatial context of the surrounding environment,^24^, ^25^ respectively. Memories that have been learned long ago and lacking in rich detail can become well consolidated in the neocortex and, ultimately, retrieved independently from the hippocampus. However, the hippocampus remains engaged either when retrieval is of relational memories that have been recently acquired, or when retrieval requires calling forth a rich set of vivid details from personal experiences.^19^, ^26^

Although models of oculomotor control have acknowledged the use of prior experience in the guidance of eye movements, the roles of the hippocampus and MTL have not explicitly been considered. How could a memory signal drive oculomotor behavior, if not at least partly via information represented in regions of the brain critical for memory, specifically the hippocampus and/or the extended MTL?^27^ Here, we focus on the contribution of representations dependent on the hippocampus and the broader MTL regarding items (people and objects), such as the arrangement of features within an item, temporal sequences, and the relative spatial positions of items within a broader environment, on ongoing visual exploration and, conversely, on the emerging role of visual exploration in the development of lasting memory representations supported by the hippocampus and MTL. Although the hippocampus and MTL may establish representations that are nonvisual in nature, given the questions discussed below concerning the role of the oculomotor system and the pattern of gaze fixations that occur across space and time in the use and formation of memories, we focus on visual memories for which configurations of features, that is, spatial and temporal relations, may be inherent within the representations.

## Models of oculomotor control

Theoretical models of oculomotor guidance are long established and supported by empirical evidence from both humans and nonhuman primates. These models propose that the selection of a saccade target is guided by a feature‐agnostic *priority map* of visual representations formed by both stimulus‐driven and goal‐directed signals that compete for selection, whereby the competition is resolved by a winner‐take‐all mechanism.^28^, ^29^, ^30^ Goal‐directed signals considered by these models often include previous experience,^31^ expectations,^32^ and, in the case of visual search, some knowledge of target identity.^33^, ^34^ Relatively recent models of oculomotor guidance stress the influence of meaning over visual salience on a priority map; specifically, where viewers fixate on a visual scene is heavily dependent on their knowledge of the semantic content and the predicted spatial positions of that content that are inherent for that scene.^35^, ^36^, ^37^ Models of how long a viewer remains looking in a given area—the duration of gaze fixations—note the importance of top‐down cognitive influences, including task demands, in addition to bottom‐up factors, such as visual salience.^38^, ^39^ Specifically, these models propose that prolonged cognitive processing may delay or even cancel subsequent saccade initiation.^40^ For instance, processing of information that is inconsistent with prior knowledge (e.g., an octopus in a barnyard) may require additional time; likewise, fixation durations under visual search instructions are shorter than under instructions to memorize the presented scene, owing to different types of cognitive processing required by each task.^39^ In contrast to other models that consider predictions of the location and duration of gaze fixations separately, Tatler *et al*.^41^ developed a model that proposes the same underlying process for *both* where and when the eyes move. Specifically, the authors note that if the purpose of eye movements is to acquire information, then where and when the eyes move can be modeled by understanding the expected benefit in moving the eyes versus remaining in the current location for ongoing and sufficient information extraction.^41^

The neural instantiation of a priority map—composed of the representations that guide where and when the eyes move—is focused on a network of regions that include the lateral intraparietal area (area LIP),^42^, ^43^ frontal eye fields (FEFs),^44^ and superior colliculus (SC),^45^, ^46^ all of which exhibit prioritized representations of visual space and activity that is crucial for the guidance and control of eye movements.^47^, ^48^, ^49^, ^50^ A complementary network of regions that includes the dorsolateral prefrontal cortex (DLPFC), anterior cingulate cortex (ACC), and supplementary eye field (SEF) is thought to be involved in the cognitive control of saccades,^51^, ^52^, ^53^, ^54^ providing additional goal‐directed inputs to the FEF and SC.

The oculomotor literature provides strong support for roles of the LIP, ACC, DLPFC, SEF, and FEF in the guidance of oculomotor behavior via an attentional template or priority map. However, despite the acknowledgment of prior experience, meaning, expectations, and knowledge—each of which invokes the broader concept of memory—as factors that influence the attentional template or priority map, and thereby influence oculomotor guidance, there has been largely no consideration of the signal, or information, emanating from the hippocampus and MTL with respect to items and locations of space that are targeted (or to be targeted) by saccades^55^ (however, see Ref. 46). This oversight is perhaps most notable when discussing findings from visual search paradigms in which a target must be located, often within an environment that invokes particular knowledge structures or schemas in memory (e.g., a kitchen). Knowledge regarding what the item looks like and where it should be located, as well as which regions have been recently viewed or where the item was located on a previous trial, drives efficient visual exploration.^14^, ^56^, ^57^ Violation of expectations from the prior knowledge, or the inability to maintain memory for previously searched items, locations, or arrays, results in longer, inefficient visual searches.^56^

Most models of oculomotor guidance invoke a mechanism for temporarily biasing the eyes away from previously fixated locations via attentional disengagement, inhibition of return (IOR),^30^, ^58^, ^59^, ^60^ or visual working memory (VWM).^29^, ^61^, ^62^, ^63^, ^64^ Despite the conceptual differences between IOR‐ and VWM‐based processes, models of oculomotor guidance have tended to conflate the two. In many cases, IOR is referred to as a *memory mechanism*. The retention processes in existing models serve to suppress spatial locations of previous fixations in a feature‐ and knowledge‐agnostic manner. Neurally, these retention signals have been considered to be restricted to either the frontoparietal network^65^, ^66^, ^67^ or subcortical control areas.^68^, ^69^, ^70^ It remains an open question whether the memory signals attributed to IOR or VWM are supported by functions of the hippocampus; recent writings have called for further inquiry.^71^, ^72^ Regardless, traditional models of oculomotor control have not accounted for the broader collection of findings that point to a role for memory representations mediated by the hippocampus and MTL in the guidance of eye movements. Specifically, whereas individuals may not necessarily need to rely on the functions of the hippocampus and MTL to guide viewing in accordance with long‐established semantic memories (as in the work of Yarbus^3^ and Loftus and Mackworth^4^), viewing behavior that changes in accordance with recent experience^5^ or that emerges in response to a task that has high relational memory demands (including perceptual processing or visual search tasks^27^, ^73^) would seem to require the contributions of the hippocampus and MTL.

## Amnesia

Early evidence that provided a specific link between the hippocampal/extended MTL memory system and visual exploration came from findings of *altered* visual exploration in cases of amnesia.^15^ Measures derived from eye tracking were used to dissociate two competing accounts of memory function, one suggesting that the hippocampus critically supports memory for the relations among items,^7^ the other suggesting that the hippocampus has a fundamental role in conscious awareness for previously stored information.^74^ Whereas neurologically intact adults showed increased visual exploration to regions of a scene that had been altered from a prior viewing (conceptually replicating the findings from Parker; see Ref. 5), these effects of memory on viewing were absent in amnesic cases of varying etiologies, including a case in which neural damage was limited to the hippocampus proper.^75^ Critical for disentangling the two competing theories of memory was the finding that such viewing effects in neurologically intact adults were observed regardless of whether the viewers had conscious awareness of the nature of the change in the scene. Eye movements may be guided by memory, even subconsciously, and a critical feature of hippocampally mediated memories is that they are relational, rather than accessible to consciousness.^15^, ^19^, ^76^ Importantly, the observed effects of memory on eye movements in this (and other) investigation(s) of memory were not because of changes in low‐level perceptual details (e.g., luminance and contrast), as such details were held constant or controlled for in the experimental design, leaving the viewer's ongoing experience as relevant (see Ref. 2 for further discussion).

Further research expanded on these initial findings. Patterns of visual exploration indicative of long‐term memory for relations among items, such as the pairing of a face with a scene^77^ or the spatial layout of objects within a scene,^27^, ^78^ were observable in neurologically intact viewers but not in cases of amnesia. Collectively, the studies that used eye tracking to investigate the nature of memory in neurologically intact individuals and in cases of amnesia went beyond the prior work that used response modalities, such as a button press, to show that relational memory can influence ongoing behavior long before an overt response is made, and even in the absence of a traditional memory task or when the viewer has no conscious awareness of the contents of memory. Yet, such influences of relational memory on ongoing viewing were absent in amnesic cases.

**Figure 2:**
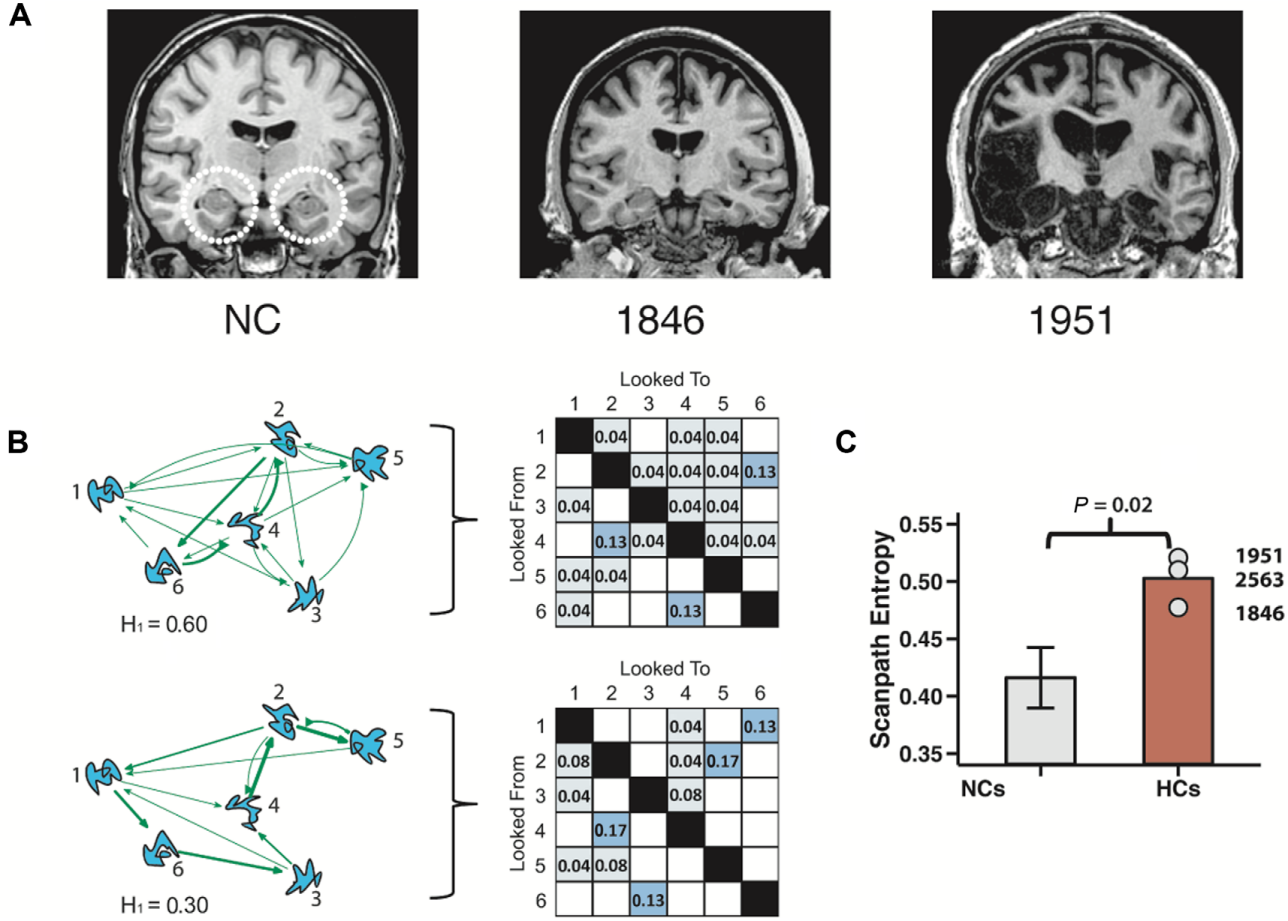
(A) Structural images are presented for a neurologically intact control participant, and two individuals with amnesia, as outlined in Lucas *et al*.^80^ The etiology for amnesic case 1846 was anoxia/hypoxia that resulted in damage confined to the hippocampus bilaterally. The etiology for amnesic case 1951 was herpes simplex encephalitis; damage was done to the bilateral hippocampus, amygdala, and surrounding cortices. Amnesic case 2563 (anoxia/hypoxia) wears a pacemaker and was unable to undergo MRI examination; bilateral volume reductions in the hippocampus were confirmed with computerized tomography. (B) Examples of relatively higher (top) and lower (bottom) entropy (H1) scanpaths. Entropy calculations were derived from transitions of fixations across regions. A higher proportion of total transitions between specific pairs of regions is noted by the thicker arrows. Transition matrices for each corresponding trial are presented on the right. Each of the scanpaths contained the same number of transition fixations. In the high‐entropy scanpath, transitions of fixations were distributed relatively evenly; the low entropy scanpath exhibits repeated sampling of fewer transition patterns. (C) The amnesic cases exhibited significantly higher levels of scanpath entropy compared with the neurologically intact control participants. Each amnesic case is noted separately, along with the group mean. Figure adapted from Ref. 80.

Relatively recent research has further shown that the amount and organization of visual exploration is altered in amnesia.^6^, ^27^, ^79^ Such changes in viewing behavior have been shown in studies for which the demands on relational memory were particularly high, even across short delays, and in studies in which there is no experiment‐imposed delay and all information is present on the display.^6^, ^27^, ^79^ Amnesic cases made more fixations and/or had an increased number of regressive fixations, compared with control participants, during difficult visual search tasks,^27^, ^73^ including one in which a multicomponent object had to be located among numerous perceptually overlapping distractors.^73^ Amnesic cases also showed higher entropy (less organization or predictability) in their viewing patterns compared with control participants when they were tasked with reconstructing the spatial locations that had been previously occupied by a set of objects with high feature overlap (Fig. 2).^80^ This is consistent with the prior work in which, unlike control participants, amnesic cases failed to show entropy differences between scenes that had either been repeated in their original form or altered from prior viewing.^6^ Such findings suggest that the hippocampus and the broader MTL may play a role in the moment‐to‐moment guidance of viewing, as lasting representations are built and used online.

Even on more traditional oculomotor tasks in which the viewer is simply required to make an eye movement to the location previously occupied by a target (memory‐guided saccade), amnesic cases with lesions that included the hippocampus and broader MTL had significantly more variable saccade landing positions than neurologically intact controls when the target location had to be held in mind over an extended delay of 20–30 seconds.^81^ Coupled with studies that showed that information within memory could guide viewing early—within the first few fixations—and long before, or even independent of, any task response that had to be made,^77^, ^82^, ^83^ this research further points to a role for hippocampally mediated memory representations in guiding viewing behavior in an obligatory and ongoing fashion.^6^ In fact, the very way in which information is viewed may be fundamentally altered with hippocampal compromise, even when there is no memory task at hand. Whereas neurologically intact adults tended to explore all the features of a face during viewing, a developmental amnesic case with congenital abnormalities to the hippocampus, fornix, and mamillary bodies showed an increased amount of visual sampling and a viewing pattern that was predominantly focused on a single face feature.^84^ In the face of compromised hippocampal function, representations of sampled information are not developed over time and do not affect ongoing viewing behavior, leading to altered viewing patterns compared with neurologically intact viewers.

Using evidence from eye tracking, debates continue to this day regarding whether conscious awareness is a fundamental feature of hippocampal‐dependent memories,^17^, ^85^, ^86^, ^87^ whether the hippocampus has a critical role in the memory for items as it does for the memory of relations among items,^88^, ^89^ and whether the information represented in the hippocampus has consequences for cognitive functions beyond memory, such as perception.^20^, ^84^, ^90^, ^91^ However, two overarching premises from this literature have achieved consensus: (1) memory representations mediated by the hippocampus and broader MTL can directly influence ongoing visual exploration; and (2) cognitive deficits caused by damage to the hippocampus and MTL can be ascertained through observable changes in visual exploration, even in the absence of any task demands that require the viewer to comment on the contents of their memory.

## Neuroimaging

Neuropsychological studies provided critical evidence for the role of the hippocampus and broader MTL in the expression of memory via eye movements. Findings from neuroimaging have provided converging evidence for the notion that memory, as mediated by the hippocampus, can be revealed via eye movements. Specifically, Hannula and Ranganath^92^ demonstrated that the strength of hippocampal activity predicts the extent to which subsequent memory‐related viewing effects were observed. Increased activity in the hippocampus during the presentation of a scene was related to disproportionate viewing of a face that had been previously paired (versus faces that had not been paired) with the scene, even when explicit retrieval of the face‐scene pairing had failed. Importantly, these findings followed a neuropsychological study using the same paradigm in which amnesic cases failed to express the eye movement–based relational memory effect, thereby directly linking the functions of the hippocampus, and the use of stored memories, to ongoing visual exploration.^77^ Similarly, across repeated viewings of configurations in a contextual cuing task in which a target had to be located among distractors, Manelis and Reder^93^ demonstrated that increased hippocampal activity predicted decreases in the number of fixations needed to locate the target. Activity in the hippocampus has also been shown to vary with activity in the frontoparietal visual attention network that, in turn, was related to strategic visual exploration during an active memory retrieval task.^94^

**Figure 3:**
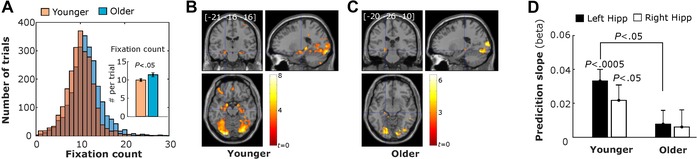
(A) Distribution of the number of fixations across trials made to novel faces for younger and older adults in Liu *et al*.^151^ Whole‐brain voxel‐wise modulation effects of the number of gaze fixations on activation in younger adults (B) and older adults (C) during viewing of novel faces (threshold *P* = 0.005). (D) Higher numbers of gaze fixations predicted stronger responses in the hippocampus for younger adults compared with older adults during viewing of novel faces, contrasted with viewing of scrambled pictures. Figure adapted from Ref. 151.

More recently, neuroimaging studies have shown that oculomotor behavior modulates neural activity in the hippocampus during perceptual processing, or encoding; thus, the relationship between oculomotor behavior and hippocampal activity is not just observed in tasks in which memory *retrieval* is required. Using simultaneous eye tracking–fMRI recordings and a scene encoding task, Henderson and Choi^95^ showed that the duration of gaze fixations was negatively related to activity strength in the hippocampus. Given the inverse relationship between the duration and number of gaze fixations when viewing time is fixed, the prediction then would be that the number of gaze fixations would relate positively to hippocampal activity. Liu and colleagues^96^ demonstrated this prediction to be correct: the number of gaze fixations made while viewing novel stimuli in a perceptual judgment task was associated with stronger neural responses in the hippocampus (Fig. 3). Moreover, greater sampling behavior during initial viewing was associated with larger reductions in hippocampal activity across subsequent viewings. Such repetition‐related decreases in neural activity (i.e., repetition suppression) have been taken as a proxy for memory formation; thus, visual exploration was related to the development of lasting representations.^96^ These findings extended prior eye‐tracking research, which showed that an increase in visual exploration predicts later memory^97^, ^98^ by suggesting that the underlying mechanism for such memory benefits is an increase in hippocampal activity.

The relationship between gaze fixations and hippocampal activity during perceptual processing (encoding) may simply reflect the amount of visual information that is extracted and, subsequently, bound into a memory representation by the hippocampus. However, on such an account, one may have expected that the metrics of fixation duration or pupil dilation, rather than the number of gaze fixations, would have been associated with hippocampal activity, as each is related to the high‐resolution inspection of visual information. As noted above, increases in fixation duration are instead related to decreases in hippocampal activity, and follow‐up analyses (work in progress, J.D.R.) revealed that pupil dilation does not have a predictive relationship with hippocampal activity. We suggest that gaze fixations, by virtue of their movements across space and time, provide additional information regarding the spatial and temporal relations among viewed elements or features, which aligns with the purported role of the hippocampus in the binding of spatial, temporal, and item‐to‐item relations.^18^, ^19^ Conceptualized in this manner, understanding the relationship between facets of oculomotor behavior and neural activity goes beyond merely noting that information is processed and instead provides clues regarding the *nature* of that information that is processed.

However, other research using a perceptual discrimination task^99^ found that hippocampal activity was not related to the overall number of gaze fixations, in contrast with findings from Liu and colleagues.^96^ Instead, when features among objects had to be maintained and compared in the moment,^99^ hippocampal activity was related to revisitations of just‐sampled regions. These seemingly discrepant findings suggest that the relationship between visual exploration behavior and hippocampal activity may be modulated by task demands and the timescale on which cognitive operations must operate. There may be patterns of viewing that reflect the engagement of the binding functions of the hippocampus to perform comparisons between complex stimuli in the moment, whereas other patterns of visual exploration, absent other cognitively demanding task operations, may reflect the formation or expression of a lasting memory representation for the global item and/or relations contained within.^6^, ^15^, ^16^, ^100^ Other aspects of viewing behavior may be linked to the bottom‐up saliency of the stimulus features and/or occur in response to the top‐down demands of the task, each of which may be unrelated to the functions of the hippocampus and does not predict the strength of its activity. The richness of eye movement data provides a powerful means to interrogate different forms of memory and cognitive states simultaneously.^16^ Further work remains to examine comprehensively the relationship between multiple metrics of visual exploration (e.g., gaze fixations, transitions into/out of distinct regions, and saccade amplitudes) and neural responses across the hippocampus and the broader MTL under a variety of task conditions to determine the type of oculomotor behavior important for the successful performance of a given task and/or the development of representations mediated by each region.

## Neurophysiology and oscillatory responses

Despite a vast literature on hippocampal and MTL function using rodent models, we have argued that research regarding neurophysiology and oscillatory responses is specifically needed in human and nonhuman primates, as it provides distinct advantages for understanding the relationship between the memory and oculomotor systems.^101^ There are marked differences in the functional organization of the memory system between primates and rodents that likely stem from ethological differences across species, with primates relying primarily on vision for guiding movements, while rodents rely on hapsis and olfaction.^102^, ^103^ Indeed, there are findings of neuronal activity in the hippocampus and MTL of primates associated with aspects of visual exploration that have not been similarly reported in the rodent.^71^, ^104^ Consequently, we focus here on research linking oculomotor behavior to hippocampal and MTL activity in primates.

In the monkey, neuronal activity (firing rate or local field potential) in the hippocampus and ERC is modulated by gaze fixations and saccades.^105^, ^106^, ^107^ Functional connectivity among different regions of the MTL, as probed by electrical stimulations, becomes stronger following a saccade, compared with time windows during which a saccade does not occur.^108^ Neuronal activity in the monkey ERC can be both the locations of fixations from multiple frames of reference^109^ and the direction of saccades during visual exploration tasks.^110^, ^111^ Likewise, in humans, the ERC responses code for gaze direction in a grid‐like fashion.^112^ Neurons in the monkey PHC and hippocampus are also responsive to the spatial locations of gaze fixations,^104^, ^113^, ^114^, ^115^, ^116^, ^117^, ^118^ likely according to allocentric reference frames.^119^

**Figure 4:**
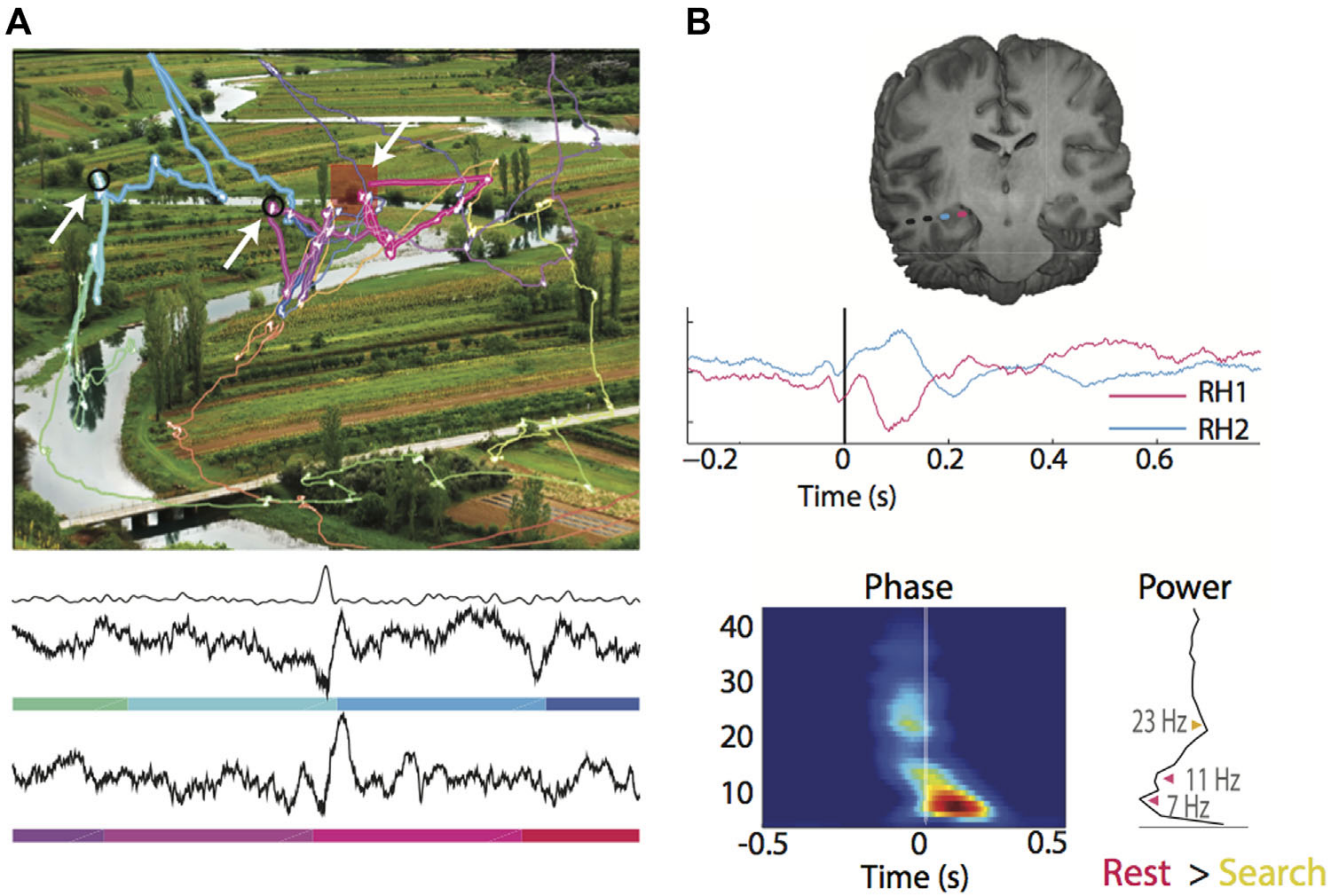
(A) Visual search task in which human and nonhuman primates in Hoffman *et al*.^120^ and Leonard *et al*.^121^ were required to detect the changing object across flicker presentations. *Top*: Representative eye movement traces overlaid onto a scene (top); the red box depicts the changing target; arrows depict the time during the eye movement search that sharp‐wave ripples were observed. Eye movement traces are color‐coded from start to the end of the trial. *Bottom*: Example segments of the recorded signal that contain ripples, in reference to the color‐coded timing of the search path. The filtered signal envelope is shown above the example segments. (B) *Top*: Localization of hippocampal depth macroelectrodes in a human patient. Average evoked responses are aligned to fixation onset. Significant deviations are observed within 200 ms postfixation and opposite‐polarity signals depending on the recording sites. *Bottom left*: Theta‐band (3–8 Hz) phase‐locking occurs for human and nonhuman primates within the 200 ms following fixation. *Bottom right*: Power in lower frequencies (6–10 Hz) is stronger during rest than during active visual search in nonhuman primates. Figure adapted from Refs. 120 and 121.

In both monkeys and humans, theta rhythm is aligned with saccades during a visual search task (Fig. 4).^120^ Hippocampal sharp‐wave ripples are also observed during visual search and may enhance the perception of foveated locations, as they are related to subsequent detection of the targets (Fig. 4).^121^ During rapid eye movement (REM) sleep, human MTL neurons have been found to increase their firing rate and synchronize their activity mainly in the theta frequency band (2–12 Hz) after REM ceases, similar to visual‐evoked responses during fixations.^122^ Theta oscillations in the MTL, and particularly in the hippocampus, have been linked to memory function in rodents, monkeys, and humans.^121^, ^123^, ^124^, ^125^, ^126^ Thus, aligning, or otherwise modifying, theta rhythm may be an important mechanism by which the oculomotor system directly influences the formation or retrieval of memories in the hippocampus or MTL.^71^, ^127^

Beyond responses in theta rhythm, work using intracranial recordings in epileptic cases, as well as magnetoencephalography recordings in healthy participants, revealed that alpha oscillations in occipital, parietal, and temporal regions, including the parahippocampus, showed significantly higher phase‐locking for subsequently remembered, versus forgotten, trials during the time just before a saccade.^128^ Work continues to comprehensively outline the broad impact of eye movements on the coordination of distinct bands of neural oscillations within and across brain regions. As noted by Rajkai and colleagues,^129^ modulation by the oculomotor system on neural responses in the hippocampus and the MTL local field can occur even in the absence of visual input;^105^, ^106^ thus, moving the eyes may serve to excite multiple occipital and temporal brain regions, across frequency bands, to facilitate efficient sensory, perceptual, and memory processing.^129^, ^130^, ^131^

Together, these findings point to a link between oculomotor behavior and the dynamics of neural responses in the hippocampus and MTL. However, despite the clear evidence that oculomotor behavior is tightly coupled with neuronal activity in the hippocampus, and that an exchange of information must exist between the oculomotor and hippocampal systems, there are no known direct (monosynaptic) anatomical connections between hippocampal subfields and regions of the oculomotor system. Thus, until recently, it was unknown how information could travel between the memory and oculomotor systems.

## Network connectivity and dynamics

**Figure 5:**
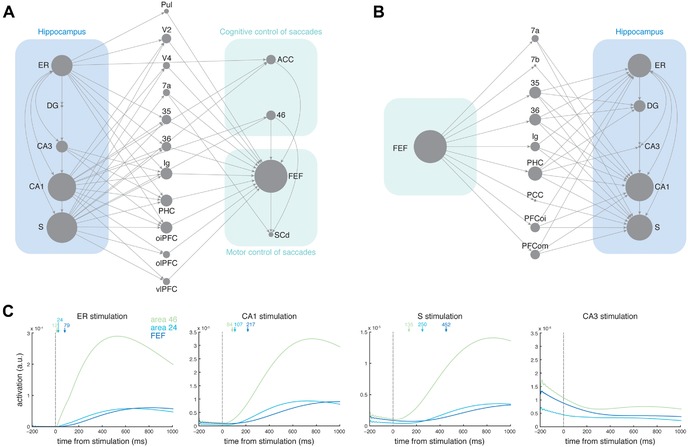
Structural connectivity between the hippocampus and the oculomotor system and its functional implications. (A) Anatomical pathways from hippocampal subregions to oculomotor areas responsible for cognitive and motor control of saccades.  (B) Anatomical pathways from frontal eye fields to hippocampal subregions. In A and B, node size is scaled for the number of shortest paths traversing each node. Only the shortest paths (disynaptic pathways) are shown. (C) Activation of oculomotor areas (46, 24, and FEF) following simulated stimulation of MTL and hippocampal subregions. Time of activation (ms) in each oculomotor area (as indicated by arrows) was determined as the time activity surpassed a baseline threshold that was defined as the mean ± 2 SD of activation for 200 ms before stimulation. No responses were observed in oculomotor areas following CA3 stimulation. Figure adapted from Refs. 101 and 134.

Work regarding the structural and functional intersections between the memory and oculomotor systems provides a comprehensive view of the vast interplay of neural regions, which may serve to guide the visual exploration on the basis of prior experiences. Using network analysis of the macaque connectome, Shen and colleagues^101^ showed that key regions in the cognitive control of oculomotor behavior, such as the DLPFC, ACC, and FEF,^51^ are among the most reachable nodes from the hippocampus. Numerous disynaptic pathways project between the subregions of the hippocampus to the FEF and to the deep layers of the SC that traverse through other MTL regions (e.g., PRC, ERC, and PHC), as well as through the extrastriate, parietal, and prefrontal cortices. Information from memory could, therefore, readily guide ongoing visual exploration through numerous structural pathways (Fig. 5A). Pathways also exist from the FEF to hippocampal subregions (Fig. 5B) so that, conversely, information regarding where saccades are directed, and when, could modulate neural responses in the hippocampus and the broader MTL, accordingly.

However, a remaining question concerned which of these pathways were *functionally* relevant, as the presence of structural connections does not necessarily equate to functional viability.^132^ Moreover, to argue that information from the memory system could reasonably influence oculomotor behavior, it would be important to show that functional responses can traverse across various anatomical pathways and, ultimately, affect neural activity within regions of the oculomotor system within the time span of a typical gaze fixation (∼250–400 ms).^133^ Although hypotheses could be generated from the knowledge of structural connectivity regarding whether activity would be relatively faster or slower via one route over another, the temporal detail (e.g., within the time of a gaze fixation) of such information required direct or modeled examination of neural responses. Using a connectome‐based model to simulate network dynamics, Ryan *et al*.^134^ examined the dissipation of activity from the memory system to oculomotor areas. Subregions of the hippocampus and regions of the MTL were each stimulated separately, and the resultant neural activity was observed as it traversed through the rest of the modeled brain. Stimulation of the CA1 field of the hippocampus, presubiculum, and any of the MTL cortices rapidly resolved into observable responses in regions of the oculomotor system, FEF, dlPFC (area 46), and anterior cingulate (area 24), well within the time of a gaze fixation (Fig. 5C). Thus, information from the memory system could reasonably affect ongoing gaze fixations through the rapid propagation of neural responses.

The hippocampus and the broader MTL mediate the formation of lasting memory representations for items, as well as their relative spatial locations and temporal orderings,^18^ which can then provide the guidance to the oculomotor system regarding precise localization of where to look and in what order.^135^, ^136^, ^137^ Multiple regions across occipital, frontal, and parietal lobes showed observable responses following hippocampal/MTL stimulation, suggesting that there is not one single region that may provide, or contribute to, the transformation of information. Moreover, signal that culminated in regions, such as V4, the superior parietal lobule, and the posterior cingulate, often appeared following observable responses in oculomotor control regions. These regions may serve to receive feedback regarding the spatial locations of foveated targets from the oculomotor system that is then integrated into representations mediated by the hippocampus and MTL.^138^, ^139^, ^140^

Additional models mimicked lesions to the network and subsequently examined the propagation of activity.^134^ Lesions to hippocampal subfields did not generally influence activity propagation from the MTL cortices, which showed quite rapid signal resolution within oculomotor regions (<100 ms). Lesions in each of the PRC, ERC, and PHC resulted in slower signal from the hippocampus throughout the network and, ultimately, to oculomotor regions. Relatively faster signal from MTL regions could result in an increase in visual exploration behavior, consistent with a case study of amnesia.^84^ Information regarding the relations among items, the broader environment, and/or the spatial organization of intra‐ or inter‐item features, as supported by the hippocampus, PHC, and ERC, respectively,^21^, ^22^, ^25^, ^141^ may be slow to develop and/or ineffective in the guidance of gaze fixations.^142^ This could result in seemingly unorganized visual exploration behavior in an effort to continually reestablish and strengthen the relations within and among items, as well as with the broader spatial configuration of the visual world.

The novel insights gained from computational modeling studies were twofold: (1) activity from the hippocampus and MTL can reach oculomotor regions within the span of a gaze fixation, and (2) hippocampal compromise speeds signal from the MTL to oculomotor regions. These findings provide a mechanistic explanation for the role of memory on active vision, and for the increased rate, or altered manner, of visual exploration in the context of hippocampal or MTL compromise, such as in amnesia. Comprehensive eye tracking and computational modeling investigations that examine whether dissociations in patterns of visual exploration and the nature of signal propagation exist depending on the location of lesion or dysfunction remain to be done. In particular, cross‐sectional and longitudinal studies of aging may provide a useful model to explore the cascade of changes in visual exploration and signal propagation that emerge with spreading dysfunction and/or structural compromise across the MTL and hippocampus.^22^, ^23^

## Aging

Older adults often show memory deficits that are similar in nature to those of amnesic cases—although not as severe^143^—and they show similar alterations in gaze patterns. Similar to amnesic cases, older adults demonstrate a decreased preferential viewing effect.^88^, ^144^, ^145^ In the preferential viewing paradigm, also termed the *visual paired comparison task*, participants are provided with repeated exposure to a set of stimuli. Subsequently, previously viewed stimuli are each presented alongside a novel stimulus. However, there is no explicit memory task provided, and there is no expectation for a later memory test; instead, viewers are asked to merely look at the stimuli presented on the screen. Increased (i.e., preferential) viewing of the novel stimulus over the previously studied stimulus is taken as indirect evidence of memory for the previously studied stimulus.^146^, ^147^ That is, if there is memory for the previously studied image, then the novel image should contain more information that is to be extracted via gaze exploration. It has been well documented that humans and nonhuman animals with hippocampal compromise show a reduced or absent preferential viewing effect, suggesting that regions of the hippocampus may be critical for the effect to be observed.^88^, ^144^ A decline in preferential viewing in aging would then seem to similarly implicate declining hippocampal function.

**Figure 6:**
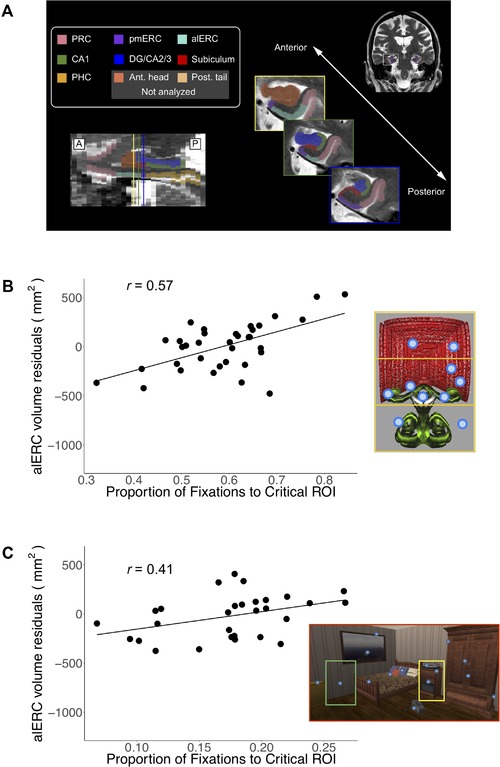
(A) Modified version of the Olsen–Amaral–Palombo segmentation protocol used in Olsen *et al*.^150^ and Yeung *et al*.^22^, ^23^ Inset images depict coronal slices of the MTL taken at various points along the long axis of the hippocampus (as shown in the sagittal view at bottom left). (B) *Left*: The relationship is plotted between alERC volume residuals (sole contribution of alERC volumes as predictors) and a viewing of the critical region of presented objects that depicted the intersection of the two object features. Larger alERC volumes were associated with greater viewing to the configurally relevant region of the objects. The relationship for viewing to recombined objects is shown; similar effects were observed for novel and repeated objects.^23^ *Right*: Example object stimulus composed of two features, with three equally sized ROIs (top, middle, and bottom) shown in yellow (ROIs not shown to participants). Gaze fixations are shown as blue circles. (C) *Left*: The relationship is plotted between alERC volume residuals (sole contribution of alERC volumes as predictors) and a viewing of the critical region of a previously presented scene that contains a manipulation from a prior viewing. Larger alERC volumes were associated with greater viewing to the manipulated region of a scene that newly contains an object (*object‐in‐place* manipulation).^22^ *Right*: Example scene stimulus is shown; manipulated regions are outlined in green (previous location of an object) and yellow (new location of an object). Gaze fixations are shown as blue circles. Figure adapted from Refs. 22, 23, and 150.

Also similar to amnesic cases, older adults do not differentially view regions of a scene that have changed from a prior viewing.^148^ Using an experimental design akin to Ryan and colleagues,^15^ in which manipulations were made to the positions of objects within a scene, Yeung and colleagues^22^ showed that increased viewing to a region that has changed from a prior viewing was also significantly correlated with volumes of the anterolateral ERC (alERC) and PHC in older adults (Fig. 6). Likewise, in a separate study, Yeung and colleagues^23^ had participants study a series of objects that were composed of two features. Subsequently, some objects were repeated in their original form, whereas other objects contained a feature swap, such that both features of the object had been previously viewed but not paired together, and other objects were completely novel. Viewing to the region that conjoined the two features, regardless of the novelty or manipulation within the object, was significantly correlated with regional volumes in the alERC (Fig. 6).^23^ Such findings were taken to suggest that the alERC may support the spatial integration of inter‐item, as well as intra‐item, features. Again, neither study required viewers to comment on the contents of their memory, as no explicit memory task was provided; thus, the eye movements provided an indirect index of mnemonic function in aging. When considered in conjunction with the findings from amnesic cases with hippocampal compromise,^15^ the findings suggest that the ERC may provide a rate‐limiting step for the binding functions of the hippocampus:^22^ information regarding spatial configurations within and across items from the ERC may be subsequently used by the hippocampus to create lasting representations regarding the broader relations among items. With ERC dysfunction, the binding functions of the hippocampus may not be fully realized. But perhaps more relevant for the present discussion, this work provided converging evidence linking specific viewing patterns to the integrity of subregions within the hippocampus/MTL that are among the first to show pathology in Alzheimer's disease,^149^, ^150^ and more generally in linking the memory and oculomotor systems.

Altered relationships between visual exploration and hippocampal activity in older adults were noted by Liu and colleagues (Fig. 3).^151^ Whereas younger adults showed a significant relationship between the number of gaze fixations and hippocampal activity, this relationship was significantly weaker in older adults. Notably, this altered relationship was observed during the first (novel) viewing of the stimuli, in a task in which no explicit memory demands were given to the participants (e.g., “judge whether the face is over/under 35 years of age”). Older adults also demonstrated a weaker relationship between gaze fixations during novel viewing and subsequent neural repetition suppression effects in the hippocampus, suggesting that older adults have difficulty in combining the spatial arrangements of features into unique, lasting representations of faces. The age‐related decline in the link between oculomotor behavior and hippocampal activity was observed despite the fact that older adults made *more* gaze fixations than the younger adults.^151^

Older adults often display increased visual sampling behavior (i.e., number of gaze fixations and number of regions sampled) compared with younger adults,^98^, ^152^, ^153^ as well as increased rehearsal of visual information through their eye movements.^154^ The number or pattern of gaze fixations has been shown to be predictive of subsequent recognition,^98^, ^154^ and restricting visual exploration during encoding can hinder subsequent memory.^155^ Further research is needed to fully characterize the causal relationship between visual exploration and hippocampal dysfunction. Specifically, questions remain regarding whether changes in visual exploration, such as those seen in aging, are merely a behavioral reflection of hippocampal dysfunction, such that increased sampling behavior is a marker of the increased effort required to properly extract sufficient information and support the development of a lasting representation, or whether altered viewing patterns reflect a (perhaps unconscious) attempt to leverage the oculomotor system to upregulate, and compensate for, a declining hippocampal system. When considering the findings from Liu and colleagues^151^ that showed an age‐related *increase* in visual exploration in the face of *decreased* hippocampal engagement, we suggest that eye movements not only passively reveal the contents of memory, but they may also be a mechanism for *actively* supporting memory encoding and retrieval, mediated through the vast neural architecture that connects the two systems.^101^, ^151^, ^156^, ^157^

## The purpose of eye movements

The above evidence from humans and animal models across behavioral, neuropsychological, neuroimaging, and computational modeling studies on characterizing visual exploration converges to suggest that there is an intimate connection between the memory and the oculomotor systems. But what is the purpose of the pervasive structural and functional intersections between the two systems?

As noted earlier, there is a consensus that eye movements are drawn to salient regions of the visual world and thereby provide the means with which to explore novel or informative areas.^3^, ^4^ Through movements across space and time, eye movements may be an outward manifestation of the hippocampal binding process.^137^ That is, saccades and gaze fixations serve as a mechanism to bind distinct elements into a coherent and lasting memory representation.^96^, ^151^, ^158^ These ideas harken back to early eye tracking research.^4^ In considering the findings from their octopus‐in‐the‐barnyard study, Loftus and Mackworth^4^ posited that since gist‐level information can be extracted early from a scene, often within a single fixation,^159^, ^160^ the purpose of the subsequent gaze fixations may be to verify whether the presented information is already contained within existing knowledge structures or schemas. On this view, longer durations of gaze fixations would reflect the time required to link or update the relevant schema with the new information (e.g., octopi *can* live on farms).^41^ That is, greater viewing is directed at the areas of interest within the environment that are not well represented within, or violate the expectations from, a viewer's schemas, and this occurs for the purpose of continually forming new memories and updating knowledge structures. This notion is supported by combined eye movement–ERP findings in which longer gaze durations were directed to, and larger neural signatures indicative of semantic processing preceded and followed the initial fixation on, a target object that was inconsistent with the meaning of the scene, compared with a consistent target object.^161^

There is also a reasonable consensus that the pattern of visual exploration is, at the very least, influenced by existing memories.^2^ Whether eye movements have a functional role at retrieval, or the influence of memory on the pattern of visual exploration at retrieval is merely epiphenomenal, is an open question.^162^ We have proposed that eye movements play a functional role at retrieval by generally reinstating the broad spatiotemporal encoding context in accordance with task demands and available cognitive resources (*gaze reinstatement*).^157^ That is, eye movements are not simply another example of an effector system that passively reflects the outcome of memory retrieval, such as is the case with a button press or verbal response. Rather, eye movements may fundamentally contribute to the retrieval of information as it unfolds.

As reviewed in more detail by Wynn and colleagues,^157^ evidence shows that viewers tend to recapitulate the spatial locations and temporal order of encoded content during subsequent viewings, including during recall of memories in the absence of visual input (i.e., looking at nothing),^136^, ^163^ when memory is merely being searched, and even during internally generated thought or problem‐solving.^164^, ^165^ The manner by which such gaze reinstatement occurs—that is, repeating patterns of visual exploration across space and time—in turn, facilitates access to, and reactivation of, associated details from memory.

Gaze reinstatement thus provides a means by which *pattern completion* of bound information in memory is retrieved in response to a partial cue.^77^, ^166^, ^167^ The information extracted via initial visual exploration may cause neural regions to engage in processes that promote the retrieval of associated details.^168^ This information then likely provides a set of predictions or expectations for the priority map of oculomotor control to guide further sampling behavior, causing an iterative, continuous cycle of gaze and memory reinstatement.^169^ Note that the notion of gaze reinstatement put forth by Wynn *et al*.^157^ suggests that it may not occur in all cases of memory retrieval and/or be related to memory performance, and indeed, gaze reinstatement is not observed in all paradigms, nor does it necessarily relate to memory performance in every instance (see Ref. 157 for review). Specifically, gaze reinstatement may aid in memory retrieval when the demands of the task exceed available cognitive resources (i.e., when information cannot be held online or within the confines of working memory) and/or when performance critically requires access to hippocampally mediated relational memory.^154^ As an example, when multiple elements, each of which is composed of a constellation of features, must be bound and retrieved across space and time, gaze reinstatement may be beneficial and related to performance, whereas retrieval of a single item with few features and occupying a single location may not require gaze reinstatement.^170^ Likewise, in cases of presumed hippocampal dysfunction, such as in aging, increases in visual exploration and increases in gaze reinstatement may support memory retrieval on simpler tasks for which younger adults either do not show gaze reinstatement or do not show a relationship between gaze reinstatement and memory performance.^154^ However, comprehensive research that examines the boundaries of gaze reinstatement—the conditions under which it is evident and the types of memory performance it supports—remains to be done.

Additionally, further research is required to determine whether gaze reinstatement contributes to the phenomenological *experience* of memory retrieval, above and beyond its contribution to the mere access of stored details. That is, gaze reinstatement, by virtue of its recapitulation of patterns across space and time, may give rise to what Endel Tulving described as *autonoetic consciousness*: the ability to transport oneself through space and time in order to call forth details from memory.^171^ In a similar manner, autonoetic consciousness can transport an individual forward in time to generate novel simulations of future events, an ability that is dependent, at least in part, on the hippocampus.^172^, ^173^, ^174^, ^175^, ^176^ On this view, gaze reinstatement may occur for multiple, distinct prior experiences or previously learned elements, which are then uniquely recombined across space and time through further visual exploration to aid in future imaginings. These views are speculative and remain to be tested.

A critical question, or test of the purported role of gaze reinstatement in memory, and even in future imaginings, is whether individuals who have compromised oculomotor function or lesions within the oculomotor network have concomitant deficits in the type or quality of memories that are formed and retrieved, or in the simulations that are generated. On the one hand, disturbances to the oculomotor system may have only minor impact on the development and use of hippocampal‐dependent relational memories, as individuals with partial visual field deficits may compensate by moving their head and body in order to foveate relevant information, and to encode the requisite spatial positioning and temporal orderings. Likewise, although information from the visual system may dominate the contents of memory in humans and nonhuman primates, hippocampal memories are composed of information gleaned from the different senses that traverses through distinct cortical processors. Nonetheless, a few neuropsychological case studies have noted deficits in oculomotor control and memory as a result of medial thalamic insult.^177^, ^178^ Also, there is evidence that oculomotor disturbances, such as those seen in progressive supranuclear palsy (PSP), were associated with reduced efficiency in visual search^179^ and reduced spatial memory span^180^ for the orientation (vertical versus horizontal) in which saccades were affected. To the best of our knowledge, paradigms that examine memory performance over extended delays have not been done with PSP cases. Work remains to link oculomotor dysfunction and/or lesions to regions within the oculomotor network to disturbances in hippocampal and MTL dynamics (e.g., propagation of neural signal and alignment of theta cycles with gaze fixations) and, ultimately, to the formation, use, and experience of memory.

## Applications of research linking memory to visual exploration

Evidence of memory as expressed through eye movements has a number of applications, spanning issues related to law enforcement (e.g., detection of concealed knowledge),^181^, ^182^, ^183^, ^184^ evaluation of expertise,^185^, ^186^, ^187^, ^188^ and development of medical training protocols.^189^, ^190^ Here, we focus on two issues related to the function of the hippocampal memory system: (1) detection of neurodegeneration, such as in cases of mild cognitive impairment (MCI) and Alzheimer's disease, and (2) cognitive therapies for the treatment of mental health disorders, specifically, posttraumatic stress disorder (PTSD).

### Detection of neurodegeneration

Eye tracking–based tasks may have predictive power for determining who is at risk of development of clinically significant cognitive decline, or who is likely to continue declining within their disease state.^191^ The aforementioned preferential viewing task has been shown to be sensitive to age‐related memory dysfunction and neurodegeneration in the hippocampus and broader MTL. The tendency to view the novel stimulus over the previously studied stimulus declines with age,^145^ and is further reduced in individuals with MCI.^192^ Preferential viewing scores predicted which older adults would progress to a diagnosis of MCI, and which individuals with MCI would progress to a diagnosis of Alzheimer's disease, within 3 years.^193^ Lower preferential viewing scores also predicted greater longitudinal cognitive decline in individuals who have mild to moderate Alzheimer's disease.^194^ Variations of the preferential viewing task have been adapted from a long‐form, laboratory‐based tasks into short (less than 5 min) screening tools that could be used in community settings.^145^, ^192^ Other variations employ a webcam, rather than an eye tracker, to score how much time the viewer spends looking at the novel versus previously viewed stimulus,^195^ much like the early work on preferential viewing with infants.^146^, ^147^ These collective findings suggest the functions of the hippocampal memory system are intimately linked with patterns of visual exploration, and that this relationship can be exploited to detect those individuals who are at risk of clinically significant cognitive decline.

However, much research remains to understand which eye‐tracking metrics and tasks would provide the earliest marker of functional change in the brain regions that are compromised first in MCI or Alzheimer's disease. Evidence from machine‐learning techniques showed that healthy control participants can be distinguished from individuals with MCI with increased sensitivity and specificity when multiple metrics of eye movement behavior (i.e., fixation duration, refixations to a previously viewed area of an image, direction of individual saccades, and pupil diameter) are used, compared with using only preferential looking times.^196^ Such findings suggest that there are a multitude of differences that may arise in the patterns of visual exploration owing to neurodegeneration,^197^ and techniques that broadly consider oculomotor metrics may be particularly well suited for distingushing healthy individuals from those experiencing significant cognitive decline.^198^

Although the field has largely focused on adaptations of the preferential viewing task to screen for neurodegeneration, this task provides a gross measure of memory function that, to date, has been associated broadly with hippocampal and MTL dysfunction. As noted earlier, on tasks that require processing and retention of intra‐ and inter‐item feature configurations, viewing behavior is related to volumes of the alERC,^22^, ^23^ which is one of the first regions to show volumetric changes in MCI and Alzheimer's disease.^149^ It is not clear whether the preferential viewing task would be a more effective screen for neurodegenerative conditions like MCI or Alzheimer's disease, compared with tasks that specifically tap into the functions of the alERC. Likewise, it is unknown whether eye‐tracking metrics would provide a more sensitive and specific screen for neurodegeneration compared with other (non‐eye‐tracking) tasks that tap into memory function. However, one advantage of eye‐tracking tasks, as specifically shown with the preferential viewing task,^145^ is that, perhaps because of their nonverbal nature and lack of overt task demands, performance is not confounded by the influences of education, language experience, or negative stereotyping around aging and memory to the same extent as more traditional neuropsychological screening tools, and therefore may be more applicable to a wider population.^145^, ^199^

To ensure the earliest possible detection of neurodegeneration, and to comprehensively track cognitive decline within an individual, development of, and direct comparison of, the specificity and sensitivity of diverse eye‐tracking tasks that tap into the specific functions of the hippocampal subfields and divisions within regions of the MTL are required. Ubiquitous screening of neurodegeneration using eye tracking is not far from realization. A number of for‐profit companies (e.g., Facebook, Google, and Apple) have acquired eye tracking–specialized firms in recent years, thus creating the opportunity for widespread integration of eye tracking into the healthcare sector.^200^, ^201^ Eye‐tracking tasks that are sensitive and specific to different types of neurodegeneration, at their earliest stages, would facilitate screening and save valuable face‐to‐face time with clinicians for those individuals who are most in need.^202^ However, it should be noted that the broader lay and medical communities are not generally familiar with eye‐tracking technology, nor with the notion that eye‐tracking tasks of memory, and even eye movement–based interventions, may have appropriate theoretical and empirical grounding.^145^, ^202^

### Cognitive therapies

Beyond the insights that measures of eye movements can bring to diagnostics, the manner by which visual exploration occurs may provide therapeutic benefits for mental health disorders, such as depression, anxiety, and, in particular, PTSD, for which decreases in hippocampal subfield volume^203^ and altered patterns of hippocampal functional connectivity have been observed.^204^ Eye movement desensitization and reprocessing (EMDR) is a treatment technique in which emotional or traumatic memories are recalled (such as in the case of PTSD), while lateral saccades are made.^205^ Altering the pattern of visual exploration, as occurs in EMDR, may, in turn, alter the amount, vividness, and/or emotional valence of the details that are retrieved from memory, and thereby alleviate debilitating symptoms.^206^, ^207^

Such findings are aligned with the aforementioned purported function of eye movements during retrieval: to reinstate the spatiotemporal context of memories.^157^ If the manner by which visual exploration occurs is important for reconstructing the details of memory, then disrupting visual exploration through repetitive lateral saccades (or stereotyped viewing patterns in general) should alter the engagement of the hippocampus and associated network, and disrupt the reestablishment of the specific spatial and temporal relations that serve as the foundation for recalling further associated details. The therapeutic benefits of alternating bilateral visual stimulation (not necessarily the movement of the eyes itself) have been associated with increased responses in the SC–mediodorsal thalamus circuit in the rodent.^208^ Other accounts suggest that altering visual exploration through lateral eye movements may alter the engagement of prefrontal cortices,^209^, ^210^ and disrupt working memory resources necessary for reconstructing details from memory, although this has been debated.^211^, ^212^

The difficulty in assessing the underlying mechanisms of these effects, as well as providing validation for the impact of stereotyped patterns of eye movements on the recall and vivid reexperiencing of details, is that most studies on EMDR do not record and measure eye movements.^213^ Recent data have shown that when participants are instructed to engage in either lateral eye movements or a working memory task, extinction is enhanced through deactivation of the amygdala and enhanced functional coupling of the amygdala with the dorsolateral–frontoparietal network and the ventromedial PFC, regions that may support the cognitive reappraisal of emotional memories.^214^ Although eye movements were recorded in this latter study, details regarding the eye movements themselves and the extent to which they varied in rate or location across conditions, or in comparison to a fixation baseline, were not provided.

EMDR provides considerable relief to those who struggle with PTSD,^213^ yet there is a lack of understanding of the mechanism of action—and hence conceptual validation—for *why* the therapy may be beneficial. Specifically, it remains largely unknown (1) the extent to which participants comply with the task instructions; (2) whether the number and quality of details recalled are directly related to changes in visual exploration, or are secondary to other aspects of the therapy protocol; and (3) which neural mechanisms are commonly related to changes in visual exploration and to the changes in the vividness and emotionality in reexperiencing prior traumatic events. Further, there are other research studies and meta‐analyses suggesting that EMDR may not provide benefits above and beyond other cognitive therapies, such as prolonged‐exposure therapy.^215^, ^216^ Again, because nearly all of EMDR research neglects to quantify eye movements, it remains unknown whether and how visual exploration differs across therapeutic conditions. Other therapies, such as prolonged exposure, may also change the nature of gaze behavior, as research has consistently documented that increasing exposure to a stimulus is accompanied by a decrease in visual exploration.^2^, ^217^ Thus, therapies like prolonged exposure and EMDR may provide benefit, as each may contain an element of altered visual exploration that, in turn, alters the number and/or intensity of details that are retrieved from memory. On this prediction, EMDR would provide benefit, but not for any of the reasons that have been suggested to date. Alternatively, it may be the case that carefully controlled studies that properly monitor and quantify eye movements still do not find a benefit for EMDR above and beyond other therapies, despite significant changes in gaze exploration across therapies, or that the underlying mechanism by which EMDR provides benefit has nothing to do with altering the pattern of eye movements. However, such research, remaining to be done, will ultimately deepen our understanding of the functional relationships between the oculomotor and hippocampal memory systems, serve to define the mechanisms that underlie the benefits garnered from each therapy, and allow for the selection of therapies to be tailored to the unique cognitive and neural profile of the individual.

## Theoretical considerations

Dysfunction in the hippocampus and the broader MTL leads to changes in the manner by which visual exploration unfolds.^2^, ^22^, ^85^ When such findings are considered alongside computational modeling research that details how neural activity may traverse the myriad of structural connections^101^ between the memory and oculomotor systems within the time of a gaze fixation,^134^ it becomes apparent that eye movements reveal the use of stored information on a moment‐to‐moment basis. The research we have reviewed here collectively aligns with theoretical accounts that suggest hippocampally mediated representations are used in service of multiple cognitive functions beyond memory, including allocating overt attention, biasing ongoing perceptual processing, and directing further actions.^104^, ^218^, ^219^ Moreover, the work in cases of amnesia, aging, and neurodegeneration presented here changes how the nature of hippocampal dysfunction can be conceived. Namely, hippocampal compromise may be both more pervasive than previously thought and result in deficits beyond memory, as it changes the very manner by which visual exploration occurs.^80^, ^84^, ^151^, ^196^

The fact that hippocampal activity is modulated by gaze fixations,^96^ and that the underlying oscillatory dynamics of the hippocampus and MTL are aligned to aspects of visual exploration,^109^, ^120^, ^219^ suggests that the operations of oculomotor and memory systems do not merely influence one another, but instead may be interdependent. This evidence regarding the reciprocal link between the functions of the hippocampal and oculomotor systems calls for a reconsideration of (1) models of oculomotor control to include the influence of the hippocampus and broader MTL and (2) models of hippocampal function to include the influence of various effector systems that govern overt behavior. Concerning the former, further research is needed to comprehensively understand how varied information from distinct neural regions are prioritized within a priority map in the guidance of oculomotor behavior.^35^ Concerning the latter, models of hippocampal function do not often consider the structural and functional intersections with the effector systems that govern overt behavior, even though decades of research has looked to changes in overt behavior (e.g., response times, actions, and gestures) to make inferences about the influence of memory.^7^, ^8^, ^220^ A paradigm shift may be needed in memory research: studying the nature of encoding or retrieval, including its underlying neural dynamics, without considering how information from the external world is integrated across movements of an effector system (here, gaze fixations), or how stored knowledge guides further exploration behaviors, may provide a limited view on how memories develop and are used. As an example of the explanatory power in considering exploratory behavior in investigations of neural function, Wirth and colleagues^118^ demonstrated that place cells in the monkey do not merely code for spatial position; rather, neurons were shown to modulate firing responses on the basis of the intersection of gaze exploration, landmark location, and goal‐oriented navigation, including the position of the self (see also Refs. 221 and 222).

## Methodological considerations

Methodological considerations arise from the work we reviewed above that links visual exploration to the formation, and subsequent use, of hippocampally mediated memories. First, neuroimaging (and accompanying behavioral) research using magnetoencephalography or electroencephalography that restricts viewing to a central fixation for the purposes of reducing eye artifacts likely muddles the resultant interpretations and generalizability of the findings.^223^ Eye movements are functional for building and retrieving memories,^2^, ^155^, ^157^, ^162^ and there is a strong relationship between visual exploration and hippocampal activity,^96^, ^224^ as well as between gaze reinstatement and neural reinstatement, more generally.^225^ The processes engaged during viewing of simple stimuli or under simple task instructions may not differ between free viewing and central fixation conditions, as the necessary information may be readily extracted from central fixation. However, reducing muscle artifacts through the restriction of eye movements may fundamentally change the cognitive processes that are engaged to support task performance when viewing multicomponent items or scenes, or when the task places considerable demands on relational memory. At the very least, maintaining central fixation may alter the engagement of the hippocampus and reduce the contribution from memory on task performance. Thus, studies that restrict eye movements during complex tasks or viewing of complex stimuli may not provide an accurate depiction of the cognitive processes that are claimed to be under study, nor of the neural regions that underlie those specific cognitive processes.

Neuroimaging studies that make interpretations regarding the pattern of neural activity may find additional explanatory power in the use of eye‐tracking metrics. For instance, as noted by Voss and colleagues,^158^ there are differences in neural responses that are tied to the complexity of presented stimuli; however, variations in stimulus complexity also invoke variations in the amount or pattern of visual exploration. Thus, neural responses may be modulated across stimulus conditions, but such modulations may simply be due to increased visual exploration. Alternatively, distinct neural responses may reflect specific aspects of visual exploration that are, in turn, related to the informational content that is either extracted from the visual world or recalled from memory during viewing.

## Conclusions

The early writings from Yarbus^3^ (p. 211) stressed the intimate link between cognition and movement of the eyes: “…people who think differently, also to some extent see differently.” Memory is one aspect of cognition that has a direct influence on the manner by which visual exploration unfolds. The last 20 years of cognitive neuroscience research from human and animal models, using behavioral, neuropsychological, neuroimaging, and computational modeling methods, has provided a wealth of information regarding the interdependence of the oculomotor and hippocampal memory systems. Previously, the functions and mechanisms of each system were considered only separately. By contrast, recent knowledge gained from evaluating them concurrently spans from mechanisms to phenomenology and includes the following.

Visual exploration serves to gather information from the environment for the purpose of forming and integrating new information into memory. The reinstatement of gaze fixations across space and time serves to recapitulate and reconstruct the rich, vivid details from memory. Functional responses within the hippocampus and the broader MTL are modulated by gaze behavior (i.e., saccades and fixations). Impairments in memory caused by damage or neurodegeneration in the hippocampus and/or MTL are readily discernable through alterations in patterns of visual exploration, even in tasks that do not have a traditional memory component or do not require conscious awareness for the expression of memory to occur. Such data show that the hippocampal and oculomotor systems interact in a reciprocal manner to influence viewing on a moment‐to‐moment basis, mediated by a vast structural and functional network of regions spanning the occipital, frontal, and parietal cortices.

Certainly, considerable work remains to comprehensively delineate the interactions between the oculomotor and hippocampal memory systems, and to understand the role of visual exploration in the experience of remembering. How previously stored information regarding items and their relations, including temporal sequences and spatial positions, are combined and/or prioritized in the guidance of gaze fixations, and what the underlying neural substrates are that support such prioritization, remain open questions. Numerous applications exist for eye‐tracking–based metrics of memory, including screening tools for neurodegeneration and therapeutic interventions for mental health disorders for which memory‐related dysfunctions are at the forefront. However, whether oculomotor indices of neural function prove to be more sensitive and specific markers for neurodegeneration, or whether they provide earlier indications of neural and cognitive decline than traditional neuropsychological tests remain to be determined. It is also unclear whether altering patterns of gaze exploration can change the detail, vividness, or phenomenological experience of memory. Similarly, the extent to which oculomotor disturbances or lesions within the oculomotor network negatively affect the type or quality of stored memory representations remains to be thoroughly investigated. Further development and refinement of eye‐tracking–based applications and therapies would benefit from increased consideration of cognitive neuroscience knowledge regarding the complex interactions between the memory and oculomotor systems.

In order to set the foundation for the above inquiries of research, we call for models of oculomotor control to consider the influence of the hippocampus and MTL on the cognitive control of eye movements, and for models of hippocampal and MTL function to consider the influence of the oculomotor system in the development and expression of memory.

## Competing interests

The authors declare no competing interests.
